# Supplementary material for: ATF4-Dependent NRF2 Transcriptional Regulation Promotes Antioxidant Protection during Endoplasmic Reticulum Stress
Source: Cancers (Basel). 2020 Mar 1;12(3):569. doi: 10.3390/cancers12030569 (PMC7139862; doi:10.3390/cancers12030569)
Supplement: Supplementary file 1 [file cancers-12-00569-s001.pdf]

Supplementary material

ATF4-dependent *NRF2* transcriptional regulation promotes antioxidant protection during endoplasmic reticulum stress

Carmen Sarcinelli, Helena Dragic, Marie Piecyk, Virginie Barbet, Cédric Duret, Audrey Barthelaix, Carole Ferraro-Peyret, Joelle Fauvre, Toufic Renno, Cédric Chaveroux and Serge N Manié.

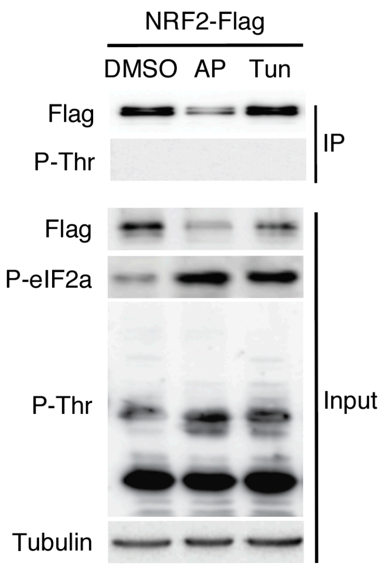

Figure S1: Analysis of phosphothreonine modification of NRF2 following PERK activation. NCI-H358 cells were transiently transfected with the NRF2-flag construct. Six hours later, cells were treated with AP (0.2 nM) or tunicamycin (0.5 ug/mL). Then NRF2 was immunoprecipitated against the flag epitope and analyzed for phosphothreonine modifications.

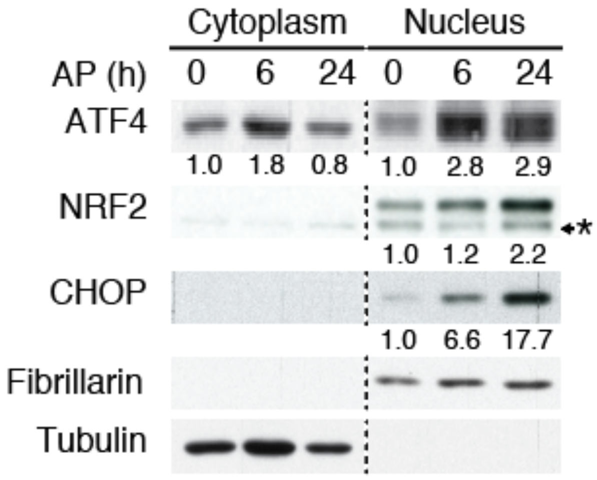

Figure S2: Kinetic analysis of cytoplasmic and nuclear amount of NRF2 following PERK induction. ATF4, NRF2 and CHOP amounts in the cytoplasmic and nuclear fractions were analyzed from NCI-H358 Fv2E-PERK cells treated with AP (0.2 nM). Fibrillarin and tubulin are provided as loading controls.

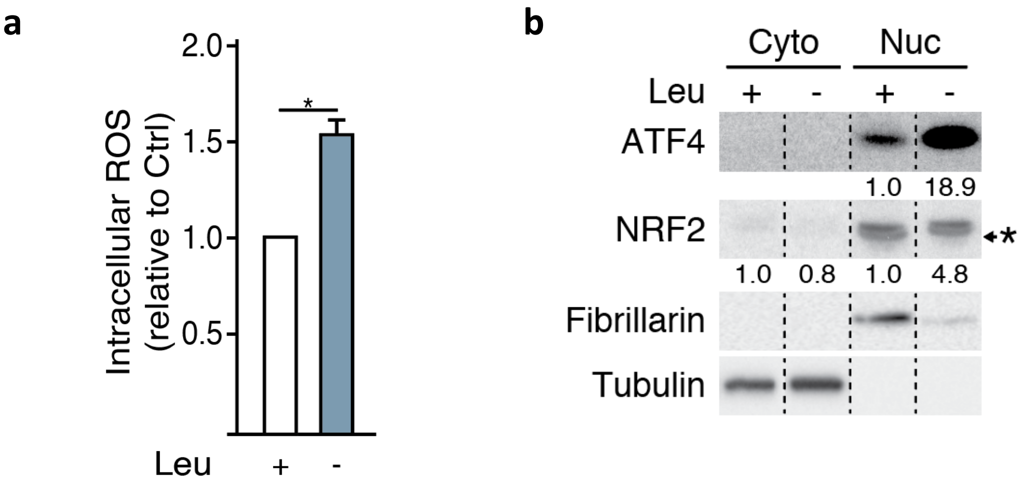

Figure S3: : a) ROS generation following leucine deprivation. H358 cells were leucine-starved for 24 hours. ROS fold increase per cell is reported. **b**) **Cytoplasmic and nuclear amount of NRF2 following leucine deprivation.** ATF4 and NRF2 amounts in the cytoplasmic and nuclear fractions were analyzed from NCI-H358 starved for 24 hours. Fibrillarin and tubulin are provided as loading controls. .

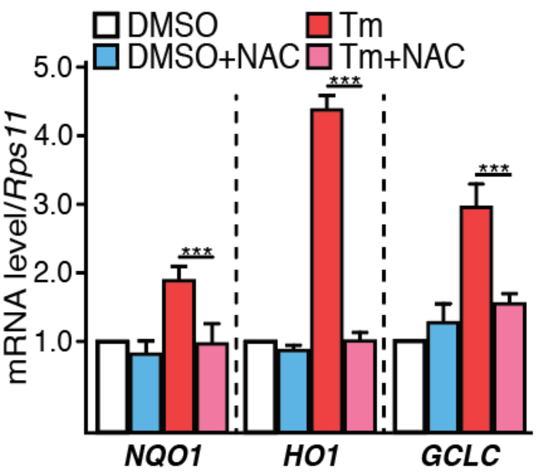

Figure S4: Expression of canonical NRF2 target genes in cells treated with tunicamycin with or without NAC. RT-qPCR analysis of NQO1, HO1 and GCLC expression levels. HBEC-3KT cells expressing were treated with or without NAC for 1 hour prior to the addition of tunicamycin for 24 hours.

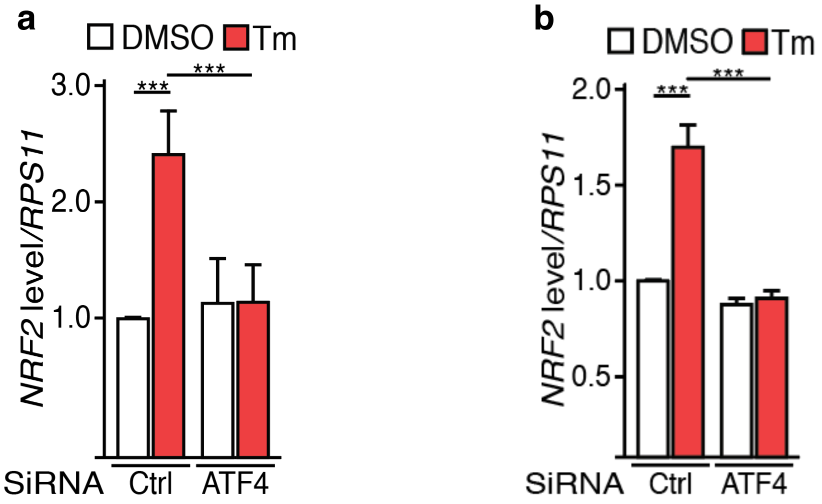

Figure S5: NRF2 expression level in cells silenced for ATF4 and subjected to tunicamycin treatment. RT-qPCR analysis of *NRF2* mRNA levels, following a 6-hour tunicamycin treatment in ATF4 silenced cells. (**a**) HBEC-3KT and (**b**) A549.

Figure 1

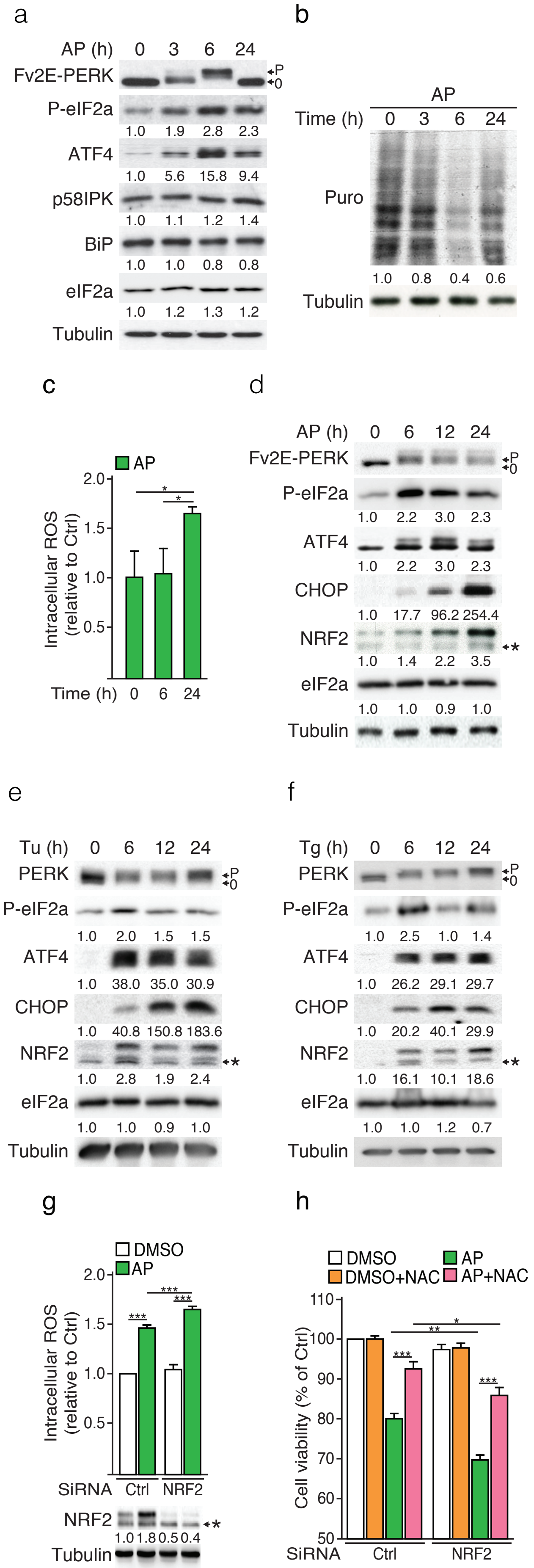
a
AP (h) 0 3 6 24
Fv2E-PERK
P
0
P-eIF2a
1.0 1.9 2.8 2.3
ATF4
1.0 5.6 15.8 9.4
p58IPK
1.0 1.1 1.2 1.4
BiP
1.0 1.0 0.8 0.8
eIF2a
1.0 1.2 1.3 1.2
Tubulin
b
AP
Time (h) 0 3 6 24
Puro
1.0 0.8 0.4 0.6
Tubulin
c
AP
Intracellular ROS (relative to Ctrl)
2.0
1.5
1.0
0.5
*
*
Time (h) 0 6 24
d
AP (h) 0 6 12 24
Fv2E-PERK
P
0
P-eIF2a
1.0 2.2 3.0 2.3
ATF4
1.0 2.2 3.0 2.3
CHOP
1.0 17.7 96.2 254.4
NRF2
*
1.0 1.4 2.2 3.5
eIF2a
1.0 1.0 0.9 1.0
Tubulin
e
Tu (h) 0 6 12 24
PERK
P
0
P-eIF2a
1.0 2.0 1.5 1.5
ATF4
1.0 38.0 35.0 30.9
CHOP
1.0 40.8 150.8 183.6
NRF2
*
1.0 2.8 1.9 2.4
eIF2a
1.0 1.0 0.9 1.0
Tubulin
f
Tg (h) 0 6 12 24
PERK
P
0
P-eIF2a
1.0 2.5 1.0 1.4
ATF4
1.0 26.2 29.1 29.7
CHOP
1.0 20.2 40.1 29.9
NRF2
*
1.0 16.1 10.1 18.6
eIF2a
1.0 1.0 1.2 0.7
Tubulin
g
DMSO
AP
Intracellular ROS (relative to Ctrl)
2.0
1.5
1.0
0.5
***
***
***
SiRNA Ctrl NRF2
NRF2
*
1.0 1.8 0.5 0.4
Tubulin
h
DMSO
AP
DMSO+NAC
AP+NAC
Cell viability (% of Ctrl)
110
100
90
80
70
60
50
*
**
***
***
SiRNA Ctrl NRF2

Figure 2

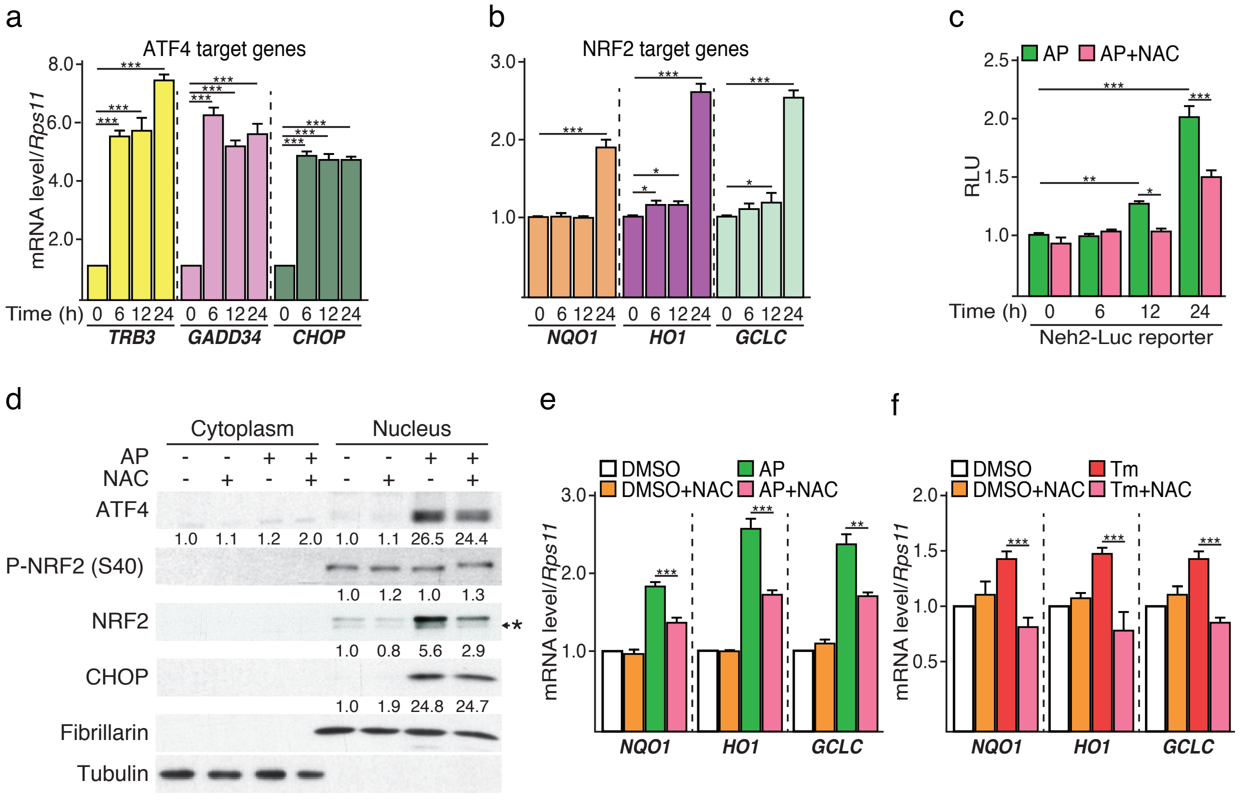

a
ATF4 target genes
mRNA level/Rps11
Time (h) 0 6 12 24 0 6 12 24 0 6 12 24
TRB3 GADD34 CHOP
b
NRF2 target genes
NQO1 HO1 GCLC
c
AP AP+NAC
RLU
Time (h) 0 6 12 24
Neh2-Luc reporter
d
Cytoplasm Nucleus
AP
NAC
ATF4
1.0 1.1 1.2 2.0 1.0 1.1 26.5 24.4
P-NRF2 (S40)
1.0 1.2 1.0 1.3
NRF2
1.0 0.8 5.6 2.9
CHOP
1.0 1.9 24.8 24.7
Fibrillarin
Tubulin
e
DMSO AP
DMSO+NAC AP+NAC
mRNA level/Rps11
NQO1 HO1 GCLC
f
DMSO Tm
DMSO+NAC Tm+NAC
mRNA level/Rps11
NQO1 HO1 GCLC

Figure 3

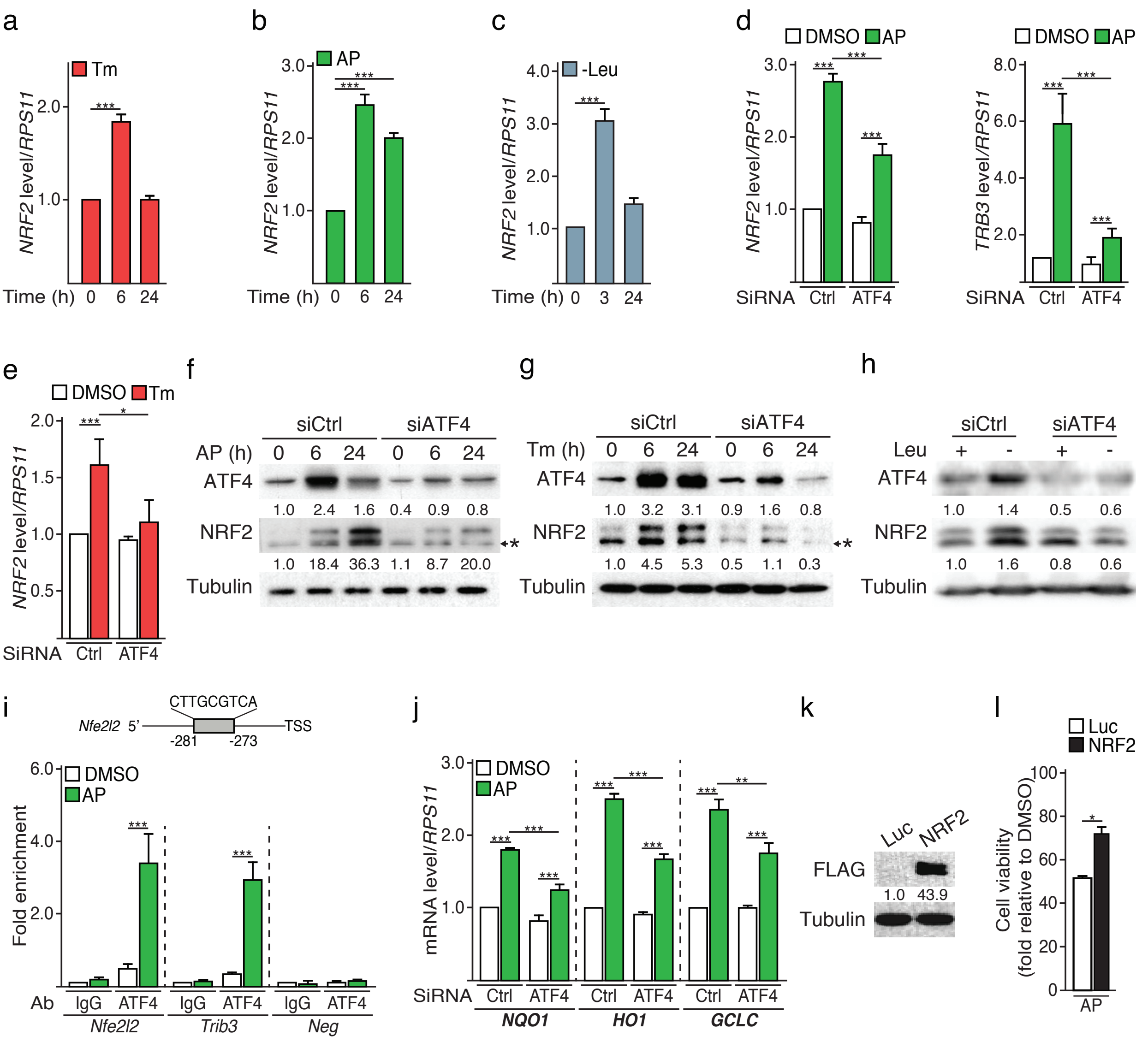
a
Tm
NRF2 level/RPS11
Time (h) 0 6 24
b
AP
NRF2 level/RPS11
Time (h) 0 6 24
c
-Leu
NRF2 level/RPS11
Time (h) 0 3 24
d
DMSO AP
NRF2 level/RPS11
SiRNA Ctrl ATF4
DMSO AP
TRB3 level/RPS11
SiRNA Ctrl ATF4
e
DMSO Tm
NRF2 level/RPS11
SiRNA Ctrl ATF4
f
siCtrl siATF4
AP (h) 0 6 24 0 6 24
ATF4
1.0 2.4 1.6 0.4 0.9 0.8
NRF2
1.0 18.4 36.3 1.1 8.7 20.0
Tubulin
g
siCtrl siATF4
Tm (h) 0 6 24 0 6 24
ATF4
1.0 3.2 3.1 0.9 1.6 0.8
NRF2
1.0 4.5 5.3 0.5 1.1 0.3
Tubulin
h
siCtrl siATF4
Leu + - + -
ATF4
1.0 1.4 0.5 0.6
NRF2
1.0 1.6 0.8 0.6
Tubulin
i
CTTGCGTCA
Nfe2l2 5' TSS
-281 -273
DMSO
AP
Fold enrichment
Ab IgG ATF4 IgG ATF4 IgG ATF4
Nfe2l2 Trib3 Neg
j
DMSO
AP
mRNA level/RPS11
SiRNA Ctrl ATF4 Ctrl ATF4 Ctrl ATF4
NQO1 HO1 GCLC
k
Luc NRF2
FLAG
1.0 43.9
Tubulin
l
Luc
NRF2
Cell viability (fold relative to DMSO)
AP

Uncropped blots corresponding to main Figure 1 panels a, b and d.

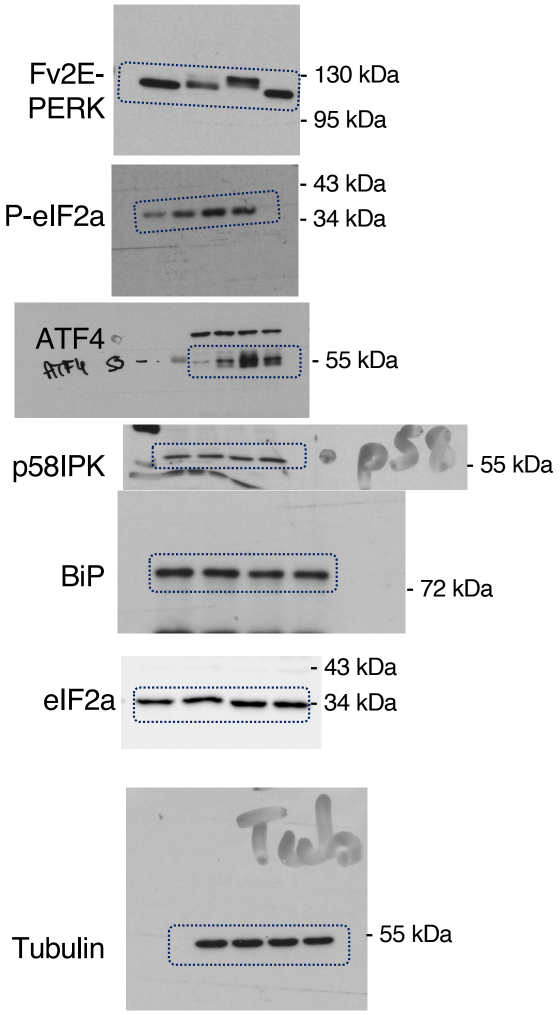

Figure 1a

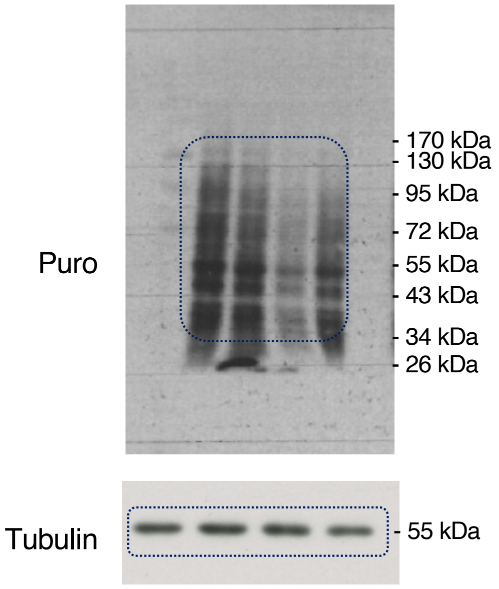

Figure 1b

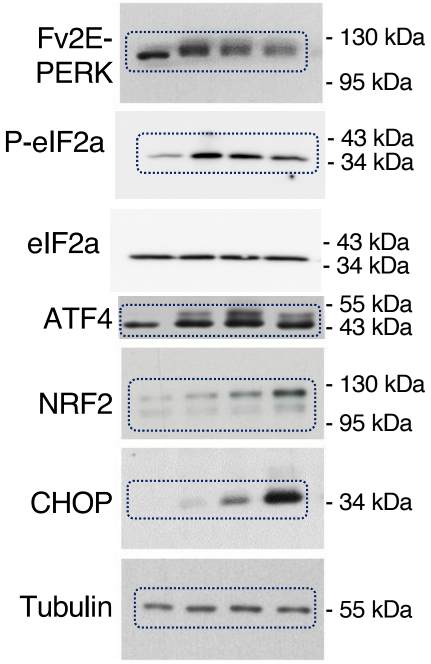

Figure 1d

Uncropped blots corresponding to main Figures 1 panels e, f and g.

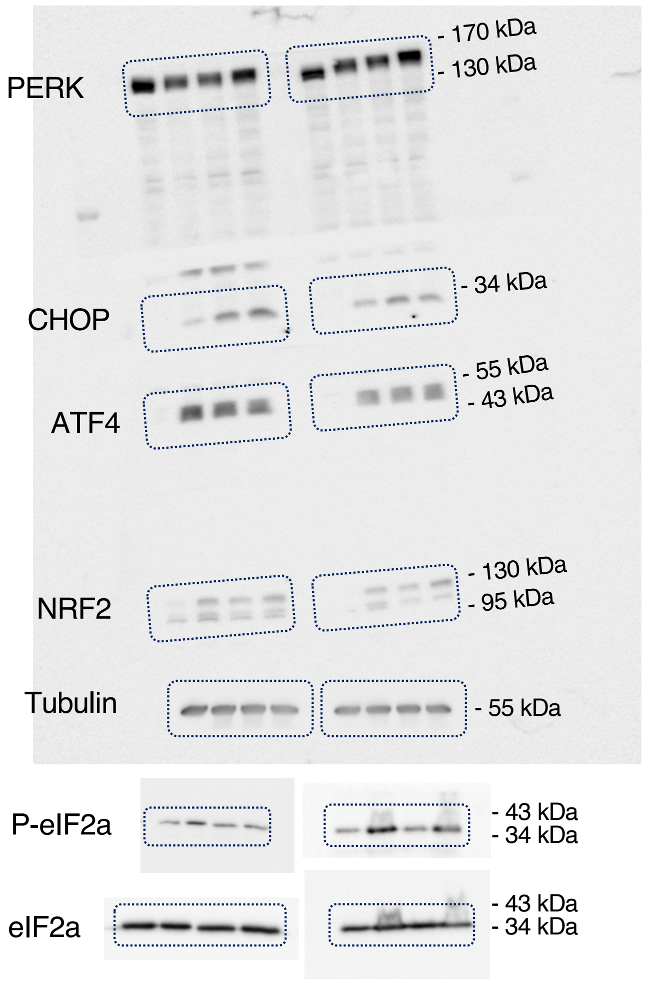

Figure 1e and 1f

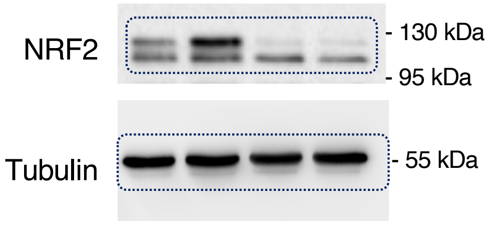

Figure 1g

Uncropped blots corresponding to main Figure 2d.

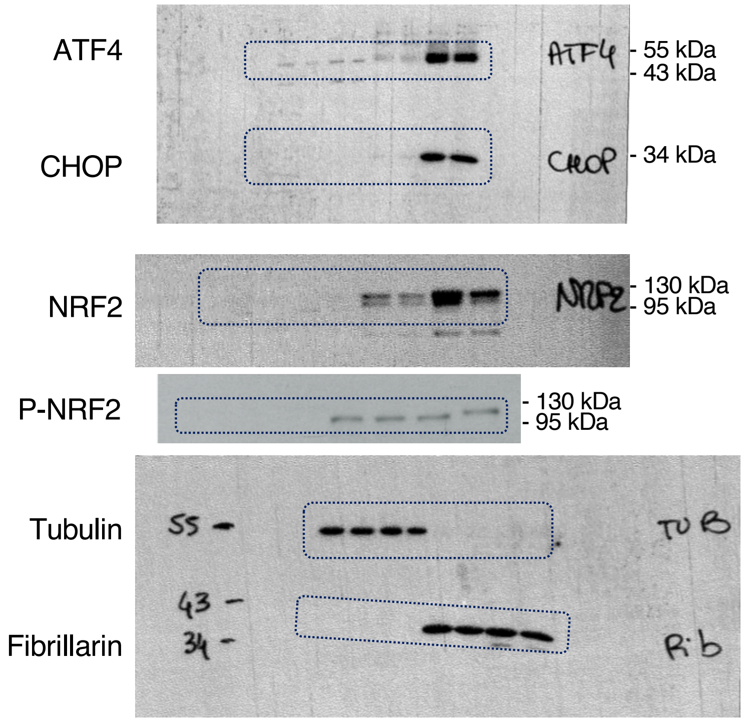

Figure 2d

Figure S4: Uncropped blots corresponding to main Figure 3 panels f, g, h and k.

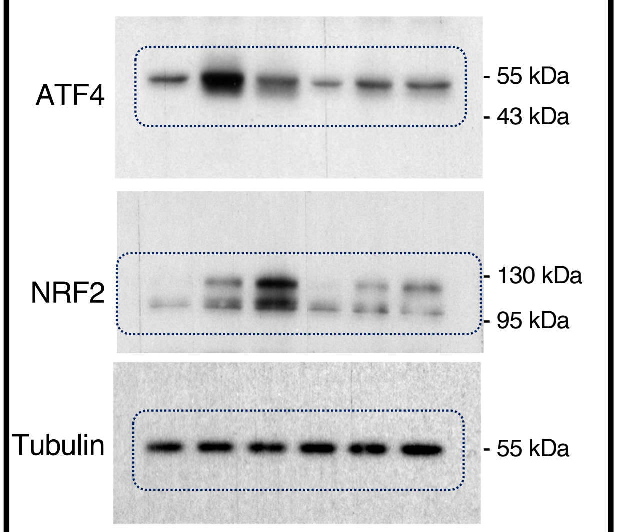

Figure 3f

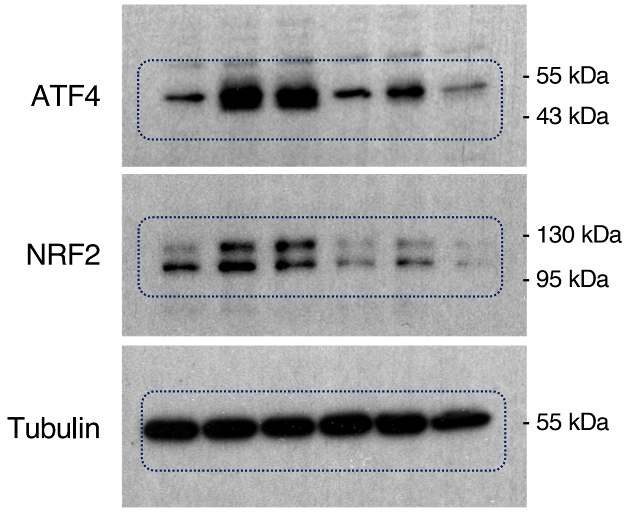

Figure 3g

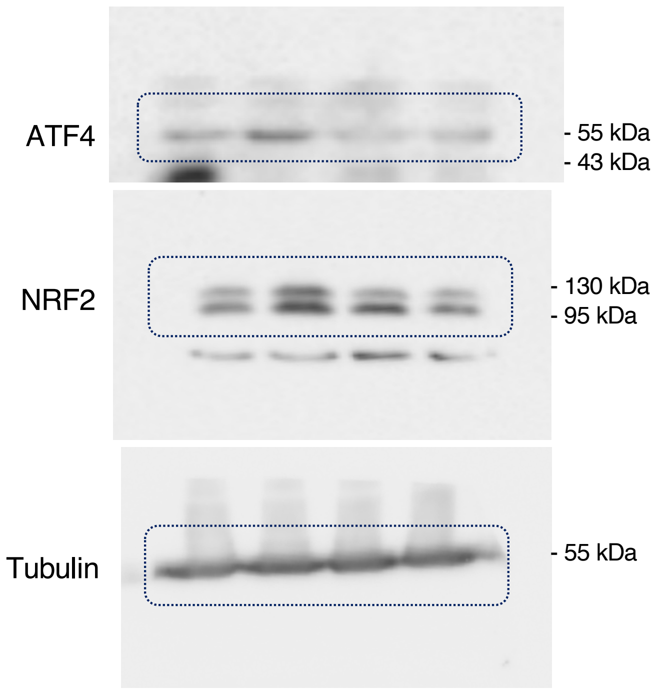

Figure 3h

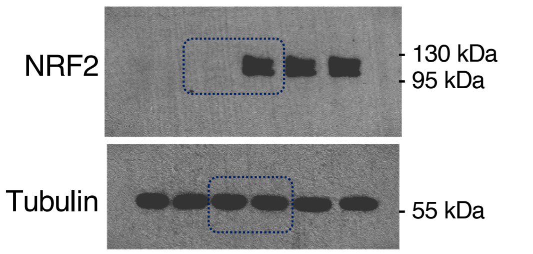

Figure 3k

Figure S3: Uncropped blots corresponding to supplemental Figure S1, S2 and S3.

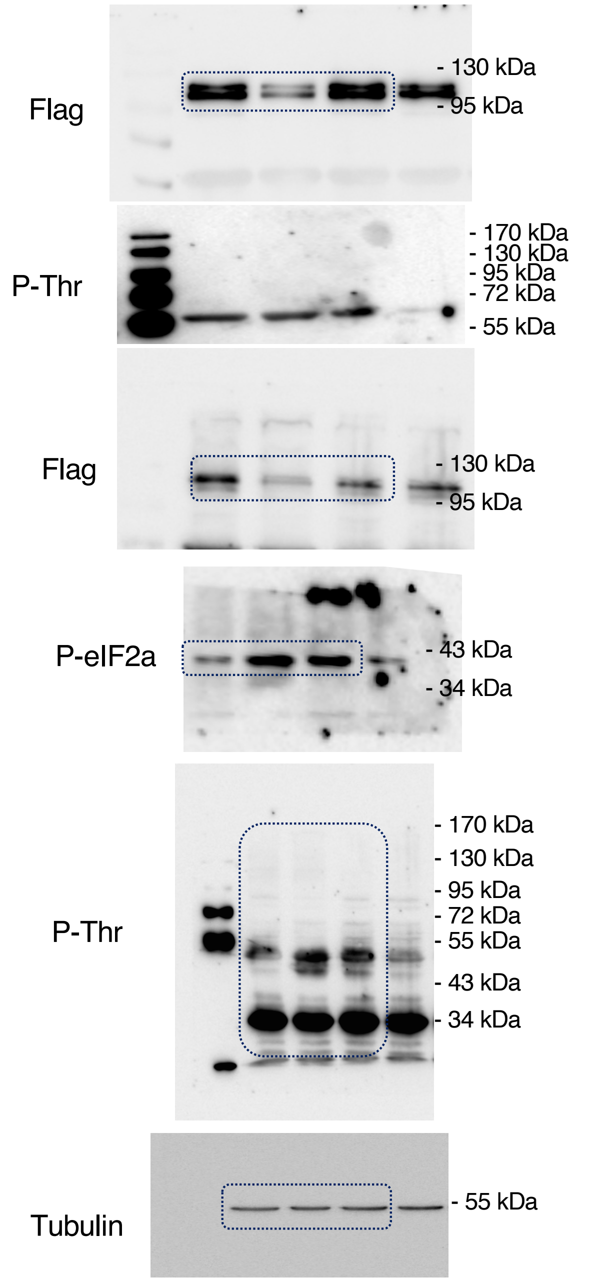

Figure S1

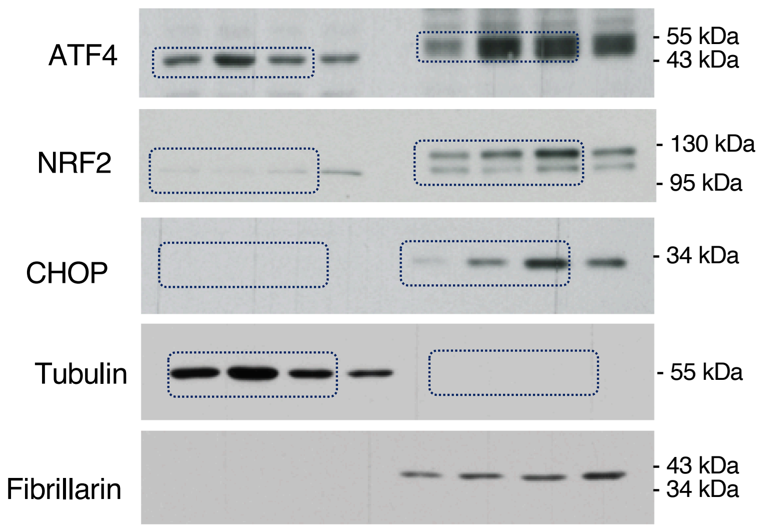

Figure S2

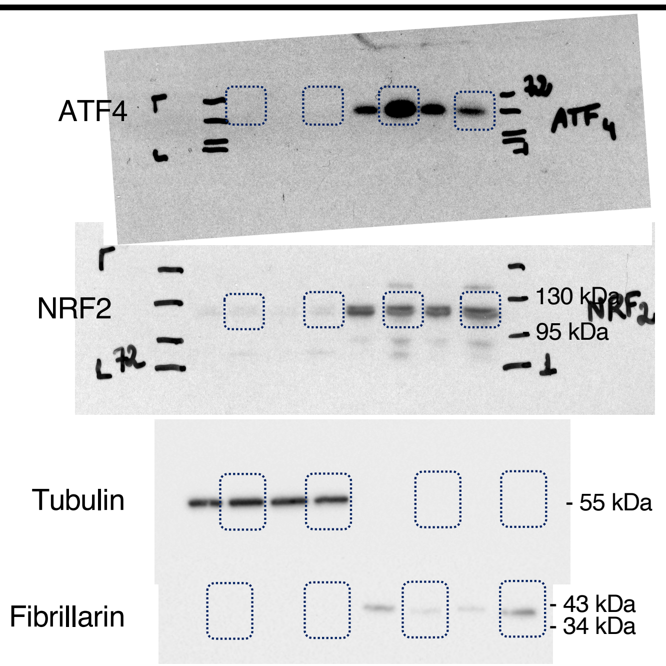

Figure S3
